# Knee-Joint Disease

**Published:** 1890-01-18

**Authors:** 


### KNEE-JOINT DISEASE.

"The Operative Treatment of Tubercular Disease of the Knee-Joint" is the title of a communication to the *British Medical Journal* of December 7th from Mr. W. Thomson, surgeon to the Richmond Hospital, Dublin. The writer says: "The knowledge that tuberculosis is a distinctly infective process, that its tendency is not only to invade neighbouring structures, but to produce distant deposits, makes it necessary that our treatment should be of a character to remove the source from which the mischief spreads." It therefore appears that Mr. W. Thomson regards tubercular joint disease as of local origin in the joint itself, for he further remarks, "whether the starting point be in the bone or the synovial membrane, the process of invasion is equally certain." Now, we regard tubercular disease of the joint as the local manifestation of a constitutional disorder, and not as a local disorder becoming constitutional. Holding the opinion he does, we are therefore not surprised to find Mr. Thomson stating that excision of the joint is the operation which gives the best promise of success. Injections, destruction of the membrane by the actual cautery, erasion, and gouging, are condemned. The arguments in favour of the latter treatment are that movement of the joint may be preserved, and that there is no subsequent shortening. But, as a matter of fact, movement is seldom if ever obtained. As regards shortening, the absence of this is not always secured, and, moreover, "our object is to save the patient's limb, and, if we have to do that at the expense of shortening, we cannot help it." On the whole we are inclined to agree with Mr. Thomson that excision of the joint is the best treatment, and this whether the disease is the result of a general constitutional taint or not, for, having once become localised, the local disease will eventually destroy the patient, if not entirely removed—exactly as is the case with cancer. The first excision of the knee-joint was performed by Crampton, in 1825, and was described in the *Lancet* of 1825, p. 29, as "scientific butchery," and the writer earnestly hoped it would be the last time such an operation would be performed, notwithstanding whatever name it may be called by the "sanguinary desperadoes" of the profession. More than thirty years afterwards, Syme inveighed against the operation. But chiefly through the exertions of Fergusson and Butcher, excision of the joint has been placed on sure ground. Mr. Thomson has had personal experience of seventeen cases, and no life has been lost. In the earlier periods of the practice of excision the mortality was greater than after amputation above the knee. During recent years the reverse has obtained. But the statistics refer to excision for all causes, not for tubercular disease alone.

